# Crystal structure of the (1*R*,2*S*,5*R*) diastereomer of acoltremon, C_18_H_27_NO_2_, from synchrotron powder diffraction data and density functional theory calculations

**DOI:** 10.1107/S2056989026006572

**Published:** 2026-06-26

**Authors:** Jacob K. Salazar, James A. Kaduk, Anja Dosen, Thomas N. Blanton

**Affiliations:** a North Central College, Department of Chemistry 131 S Loomis St Naperville IL 60540 USA; b North Central College, Department of Physics 131 S Loomis St Naperville IL 60540 USA; c Illinois Institute of Technology, Department of Chemistry, 3101 S. Dearborn St., Chicago IL 60616, USA; d ICDD, 12 Campus Blvd., Newtown Square, PA 19073-3273, USA; University of Aberdeen, United Kingdom

**Keywords:** powder diffraction, acoltremon, Tryptyr, Rietveld refinement, density functional theory

## Abstract

The crystal structure of the (1*R*,2*S*,5*R*) diastereomer of acoltremon [systematic name: (1*R*,2*S*,5*R*)-2-isopropyl-*N*-(4-meth­oxy­phen­yl)-5-methyl­cyclo­hexane-1-carboxamide], C_18_H_27_NO_2_, has been solved and refined using synchrotron X-ray powder diffraction data, and optimized using density functional theory techniques. Acoltremon crystallizes in space group *P2_1_2_1_2_1_* and the crystal structure consists of corrugated layers lying parallel to the *bc* plane. N—H⋯O hydrogen bonds link the mol­ecules into chains propagating along the *a*-axis direction, with graph-set descriptor *C*^1^_1_(4).

## Abstract 2

The crystal structure of the (1*R*,2*S*,5*R*) diastereomer of acoltremon has been solved and refined using synchrotron X-ray powder diffraction data, and optimized using density functional theory techniques.

## 1. Chemical context

Acoltremon, C_18_H_27_NO_2_ (sold under the brand name Tryptyr in the United States, and also known as AR-15512) is used to treat dry-eye syndrome. It is administered as a preservative free dilute solution in eye drops for the purpose of increasing basal tears production. The systematic name (CAS Registry Number 68489-09-8) is (1*R*,2*S*,5*R*)-*N*-(4-meth­oxy­phen­yl)-5-methyl-2-propan-2-yl­cyclo­hexane-1-carboxamide.
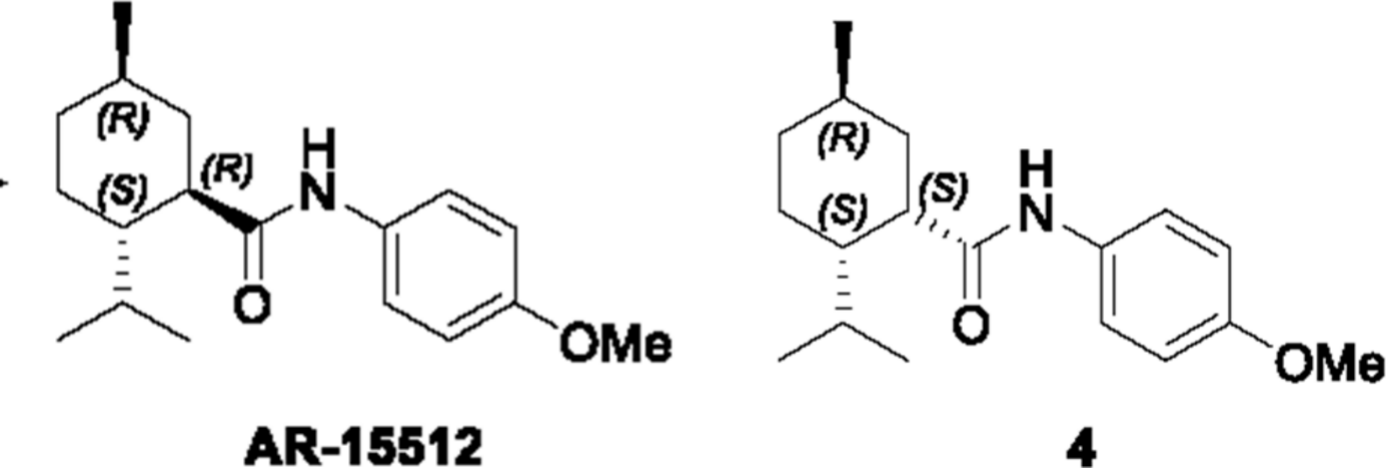


A crystal structure of acoltremon at 100 K has been reported (Rodriguez-Arévalo *et al.*, 2021 ▸), but it is of the (1*S*,2*S*,5*R*) diastereomer, **4**, and not the active pharmaceutical. We are unaware of any published powder diffraction data on the (1*R*,2*S*,5*R*) diastereomer.

This work was carried out as part of a project (Kaduk *et al.*, 2014 ▸) to determine the crystal structures of large-volume commercial pharmaceuticals, and includes depositing high-quality powder diffraction data for them in the Powder Diffraction File (Kabekkodu *et al.*, 2024 ▸).

## 2. Structural commentary

**Figure 1:**
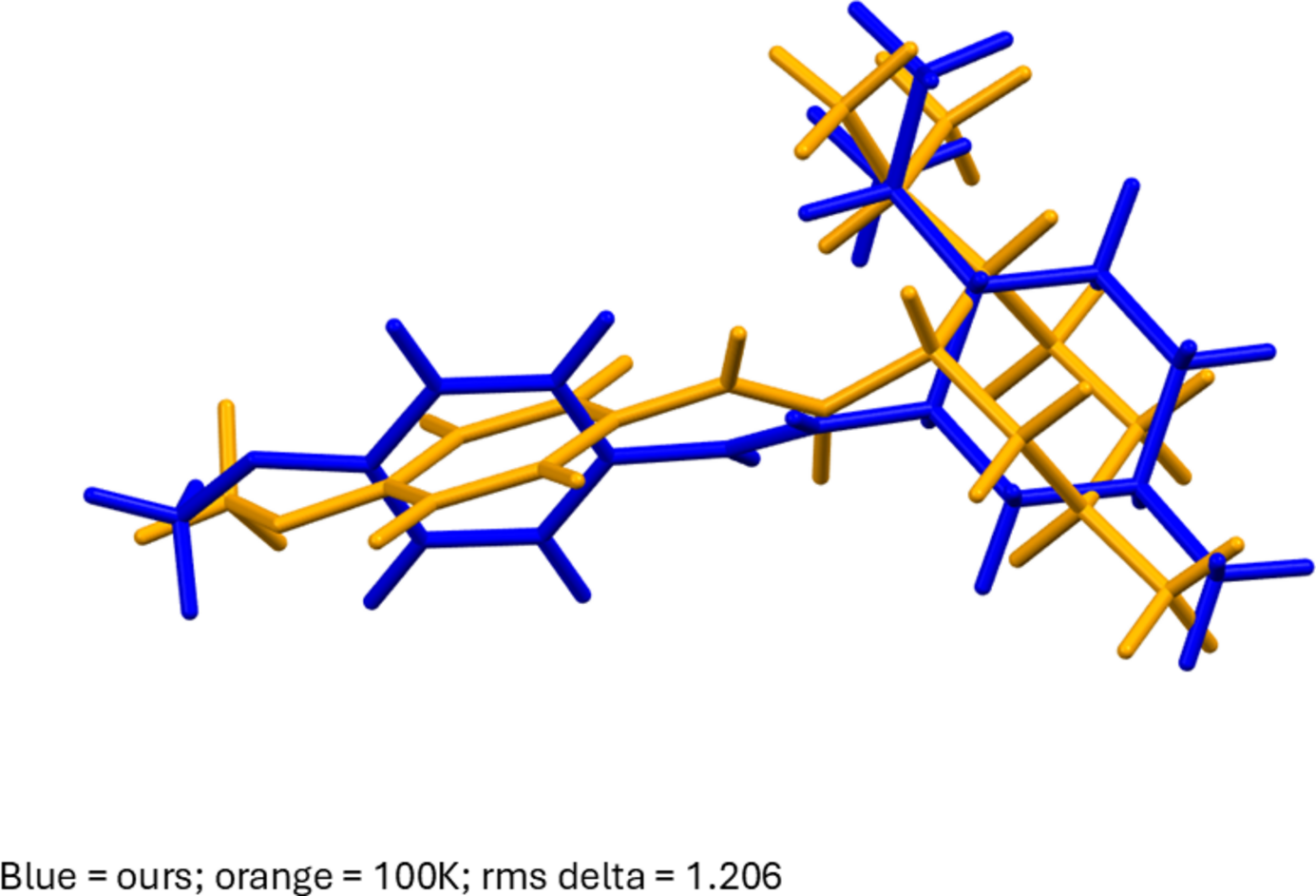
Comparison of the (1*R*,2*S*,5*R*) diastereomer characterized in this study (blue) to the (1*S*,2*S*,5*R*) diastereomer characterized by Rodriguez-Arévalo *et al.* (2021 ▸; orange). The root-mean-square Cartesian displacement is 1.206 Å.

The dispersion-corrected *VASP* calculations indicate that the structure of the (1*R*,2*S*,5*R*) diastereomer determined here is 23.3 kcal mol^−1^ lower in energy than that of the (1*S*,2*S*,5*R*) diastereomer determined by Rodriguez-Arévalo *et al.* (2021 ▸) (see Table 5 in the supporting information). As expected, the mol­ecules are quite different (Fig. 1 ▸), with a root-mean-square Cartesian displacement of 1.206 Å.

**Figure 2:**
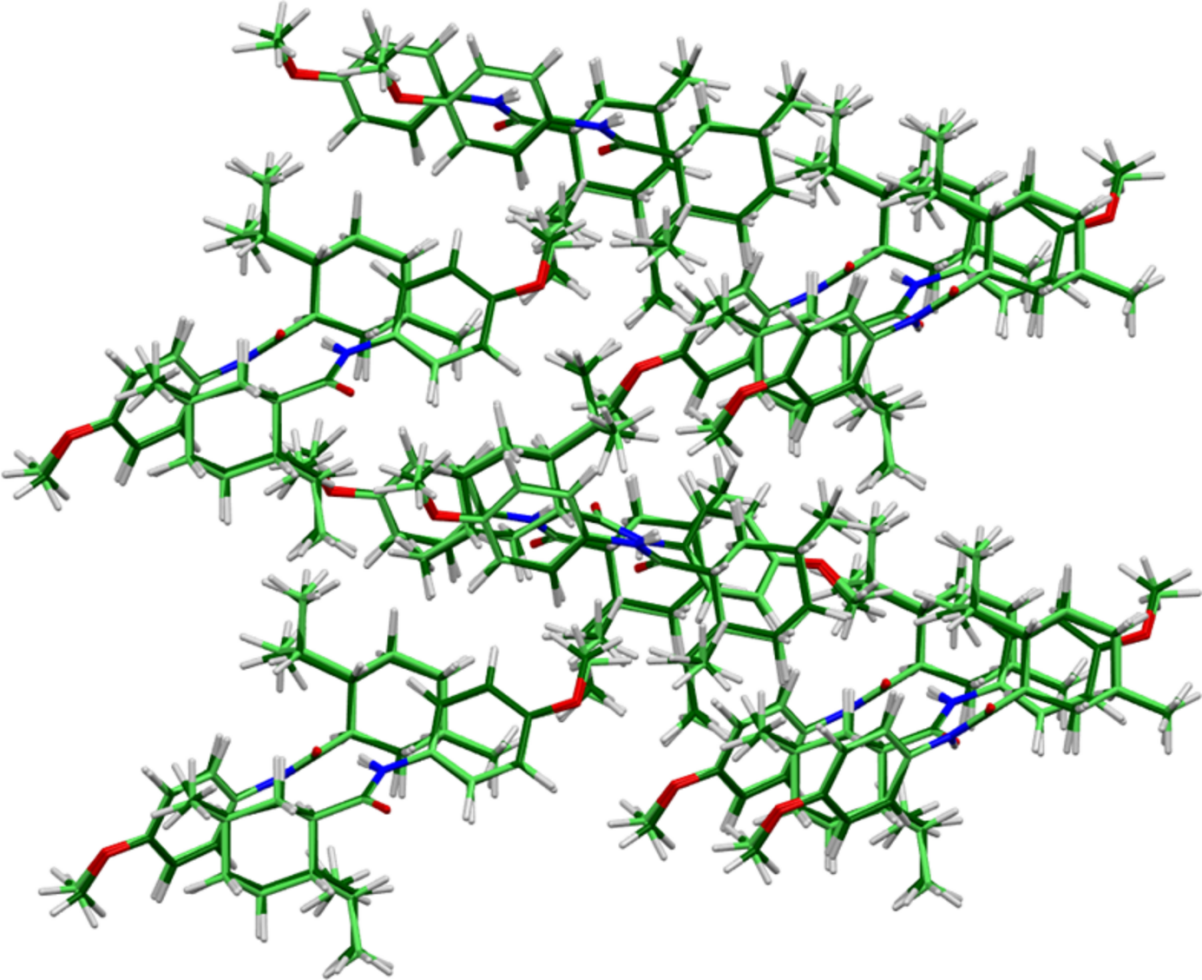
Comparison of the Rietveld-refined (colored by atom type) and *VASP*-optimized (pale green) structures of acoltremon, calculated using the *Mercury* CSD-Materials/Search/Crystal Packing Similarity tool. The root-mean-square Cartesian displacement is 0.133 Å.

**Figure 3:**
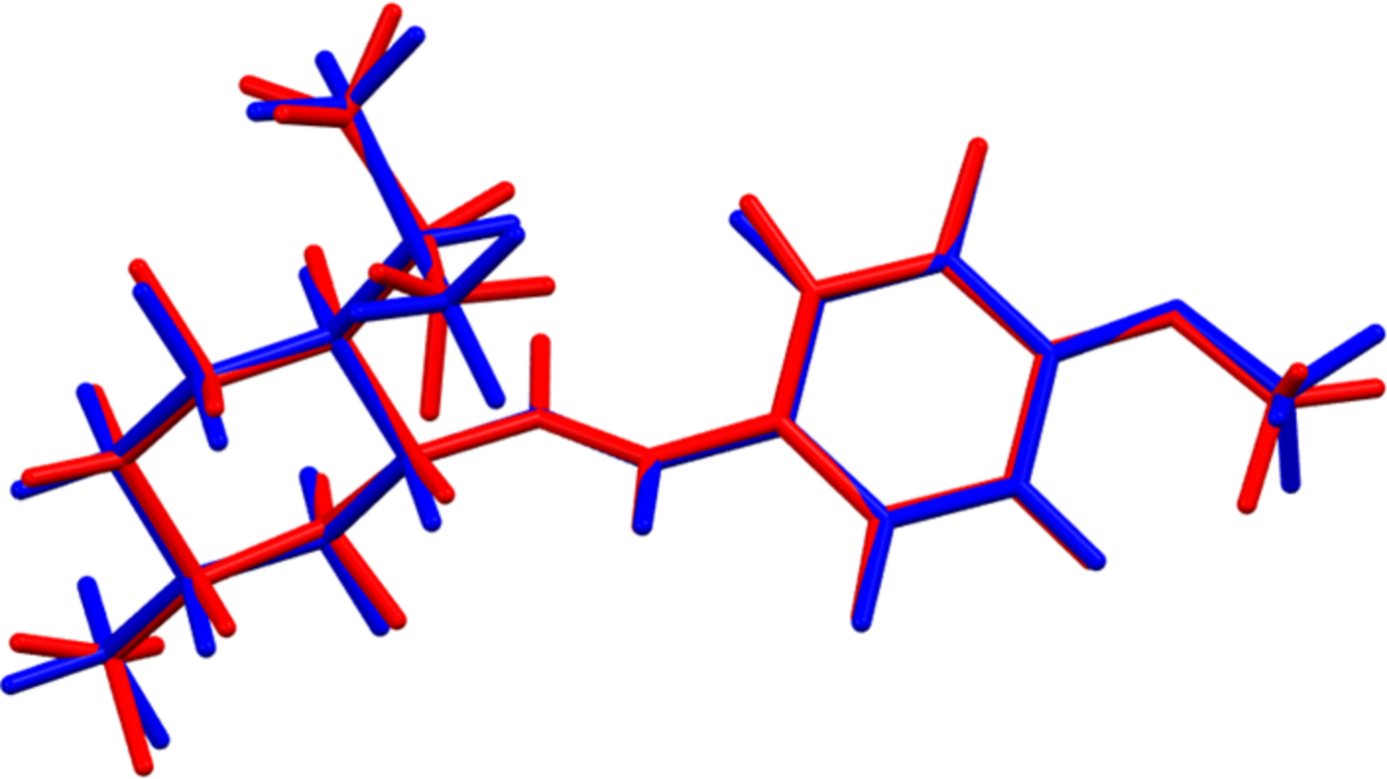
Comparison of the refined structure of acoltremon (red) to the *VASP*-optimized structure (blue). The comparison was generated using the *Mercury* Calculate/Mol­ecule Overlay tool; the root-mean-square Cartesian displacement is 0.108 Å.

**Figure 4:**
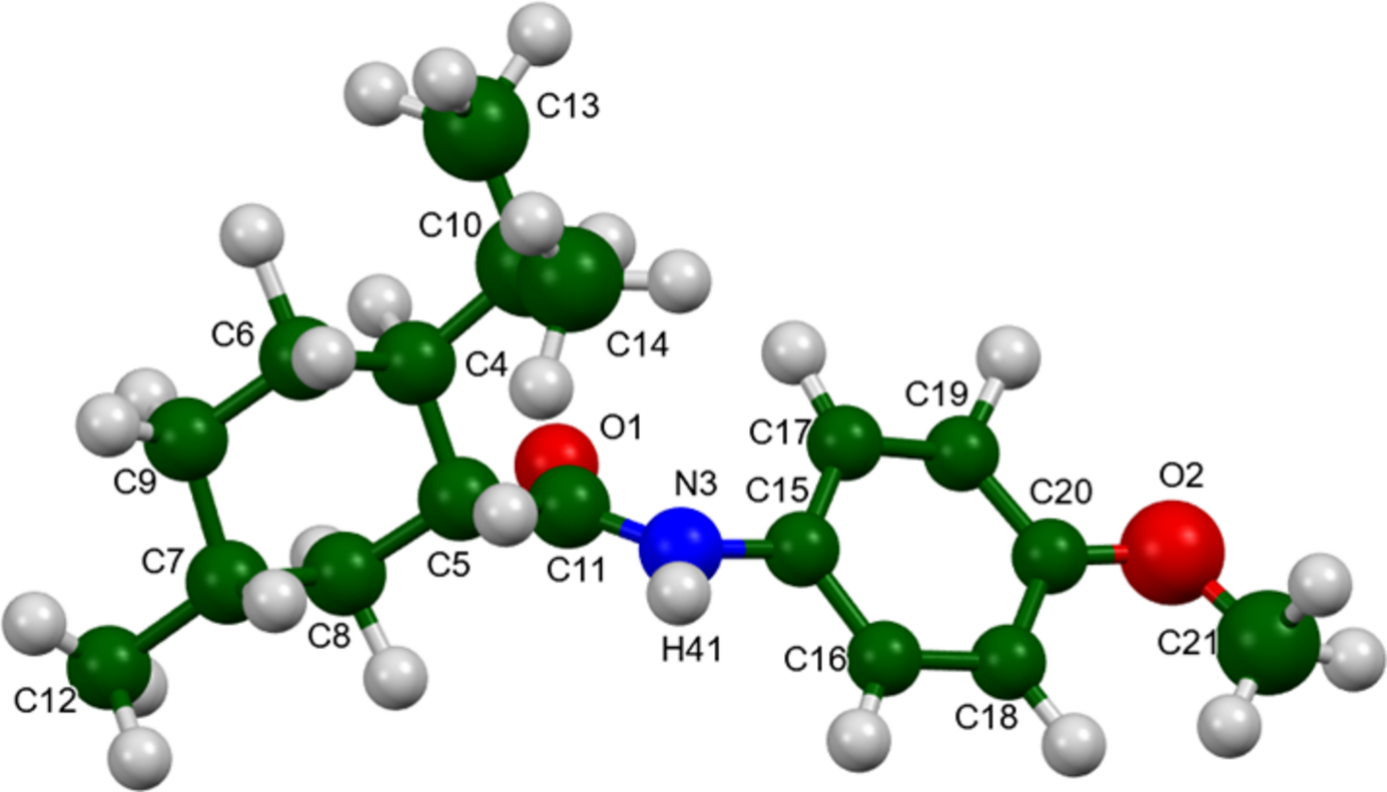
The asymmetric unit of acoltremon, with the atom numbering. The atoms are represented by 50% probability spheroids.

The root-mean-square difference of the non-H atoms in the Rietveld-refined and *VASP*-optimized structures of acoltremon, calculated using the *Mercury* (Macrae *et al.*, 2020 ▸) CSD-Materials/Search/Crystal Packing Similarity tool is 0.133 Å (Fig. 2 ▸); the structures are essentially identical. The root-mean-square Cartesian displacement of the non-H atoms in the refined and optimized structures, calculated using the *Mercury* Calculate/mol­ecule overlay tool, is 0.108 Å (Fig. 3 ▸). The agreements are within the normal range for correct structures (van de Streek & Neumann, 2014 ▸). The asymmetric unit is illustrated in Fig. 4 ▸. The remaining discussion will emphasize the *VASP*-optimized structure.

All the bond distances, bond angles, and torsion angles fall within the normal ranges indicated by a *Mercury* Mogul geometry check (Macrae *et al.*, 2020 ▸). Quantum chemical geometry optimization of the isolated acoltremon mol­ecule (DFT/B3LYP/6-31G*/water) using *Spartan ’24* (Wavefunction, 2025 ▸) indicated that the observed conformation lies 2.7 kcal mol^−1^ above a local minimum, which has a similar overall conformation (r.m.s. displacement = 0.338 Å); the difference is mainly in the orientation of the phenyl ring. Similarly, the observed conformation of the (1*S*,2*S*,5*R*) diastereomer lies 3.4 kcal mol^−1^ higher in energy than a local minimum, which differs more (r.m.s. displacement = 0.653 Å), mainly in the orientations of the isopropyl, methyl, and phenyl groups. These single-mol­ecule calculations indicate that the diastereomer of this study is 2.4 kcal mol^−1^ more stable than the other one.

## 3. Supra­molecular features

**Figure 5:**
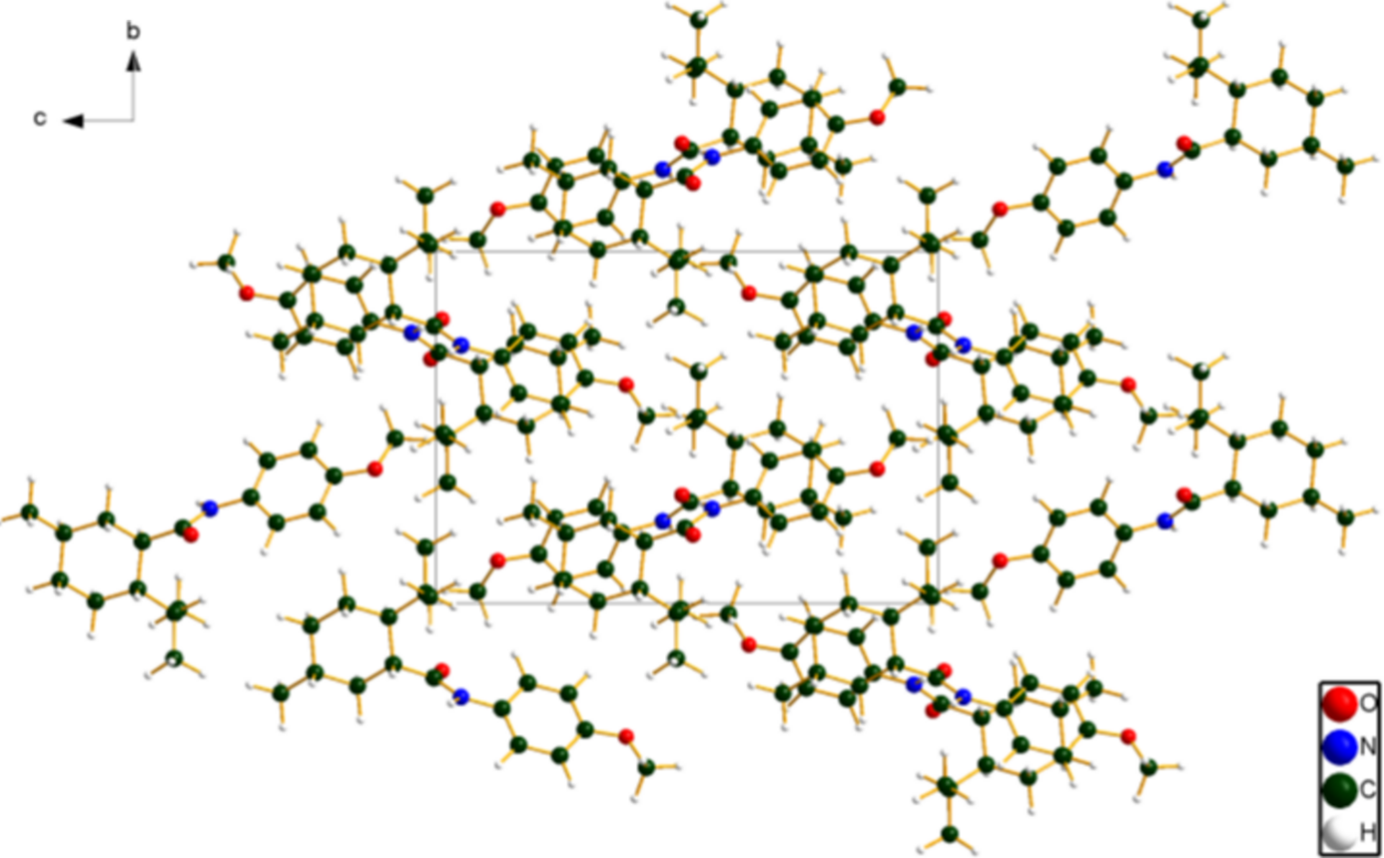
The unit-cell packing of acoltremon, viewed down the *a*-axis direction.

**Figure 6:**
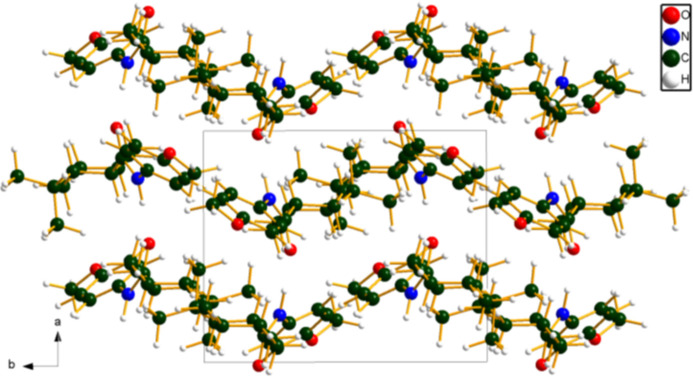
The unit-cell packing of acoltremon, viewed down the *c*-axis direction.

A view down the *a* axis of the crystal structure (Fig. 5 ▸) shows the mol­ecules clearly, but a view down the *c* axis (Fig. 6 ▸) makes it clear that the structure consists of corrugated layers lying parallel to the *bc* plane. The *Mercury* aromatics analyser indicates only extremely weak phen­yl–phenyl inter­actions, with distances ≥ 8.56 Å. The mean Miller plane of the mol­ecule is approximately (721).

Analysis of the contributions to the total crystal energy of the structure using the forcite module of *Materials Studio* (Dassault Systèmes, 2025 ▸) indicated that bond, angle, and torsion distortion terms contribute about equally to the intra­molecular energy. The inter­molecular energy is dominated by van der Waals attractions, which in this force field based analysis include hydrogen bonds. The hydrogen bonds are better discussed using the results of the DFT calculation.

**Figure 7:**
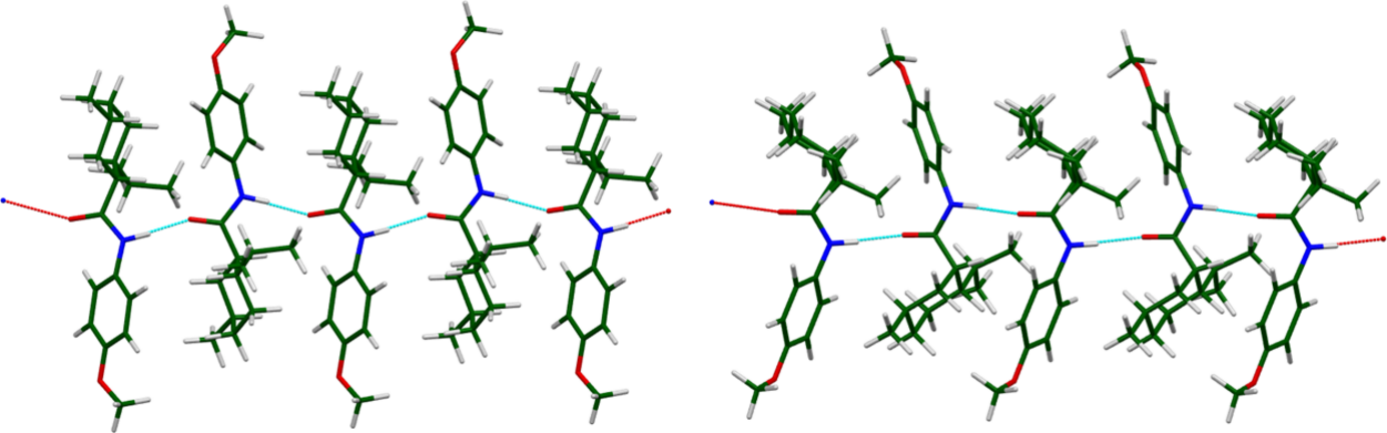
The hydrogen bond chains in the (1*R*,2*S*,5*R*) diastereomer characterized in this study (left) and the (1*S*,2*S*,5*R*) diastereomer (right) characterized by Rodríguez-Arévalo *et al.* (2021 ▸). In each case the crystallographic *a* axis is horizontal.

The hydrogen bonds are summarized in Tables 1 ▸ and 2 ▸. In both the (1*R*,2*S*,5*R*) diastereomer studied here and the (1*S*,2*S*,5*R*) diastereomer of Rodriguez-Arévalo *et al.* (2021 ▸), the amino and carbonyl groups link the mol­ecules into chains (Fig. 7 ▸) propagating along the *a*-axis direction, with graph-set descriptor (Etter, 1990 ▸; Bernstein *et al.*, 1995 ▸; Motherwell *et al.*, 2000 ▸) 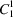
(4). These chains link the corrugated layers. However, the patterns of C—H⋯O and C—H⋯C hydrogen bonds are almost completely different between the two diastereomers.

**Figure 8:**
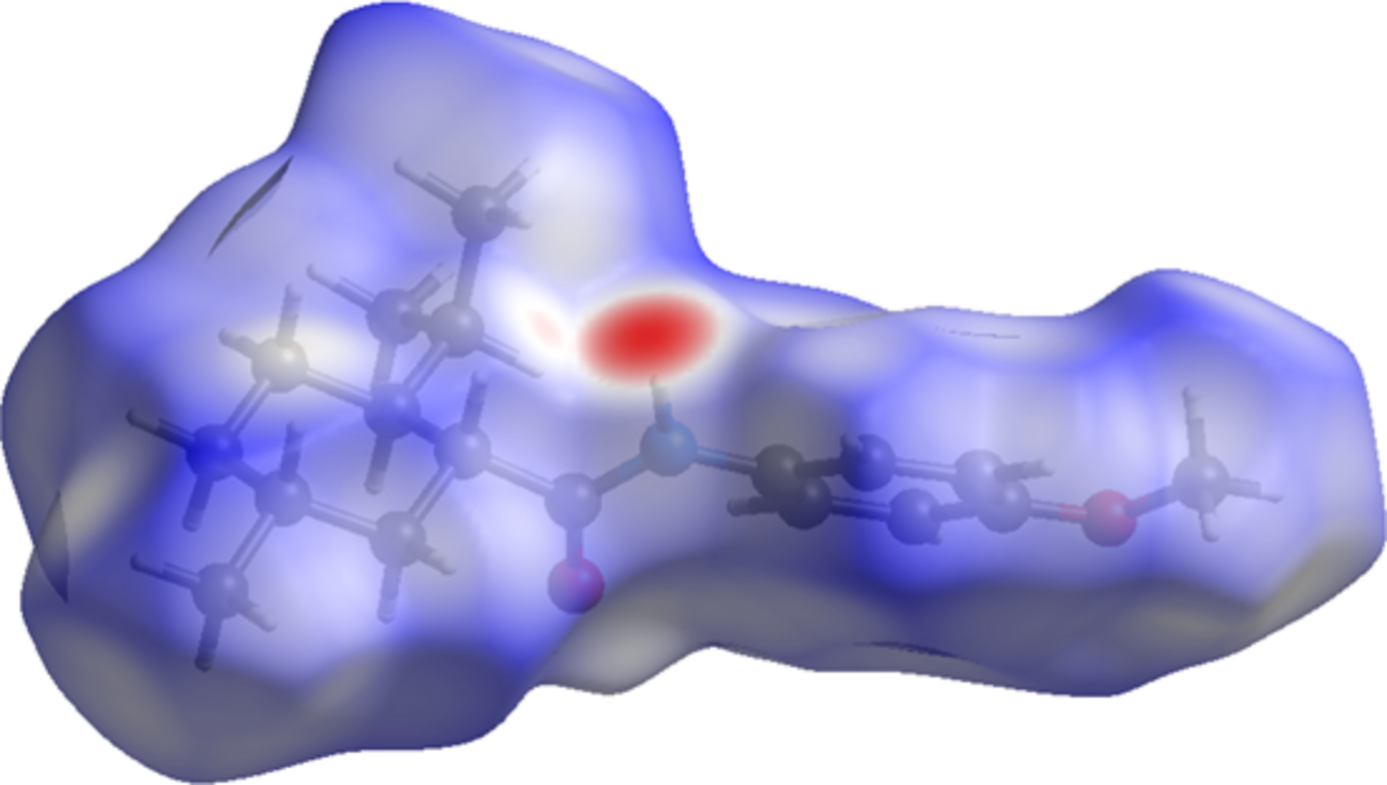
The Hirshfeld surface of acoltremon. Inter­molecular contacts longer than the sums of the van der Waals radii are colored blue, and contacts shorter than the sums of the radii are colored red. Contacts equal to the sums of radii are white.

**Figure 9:**
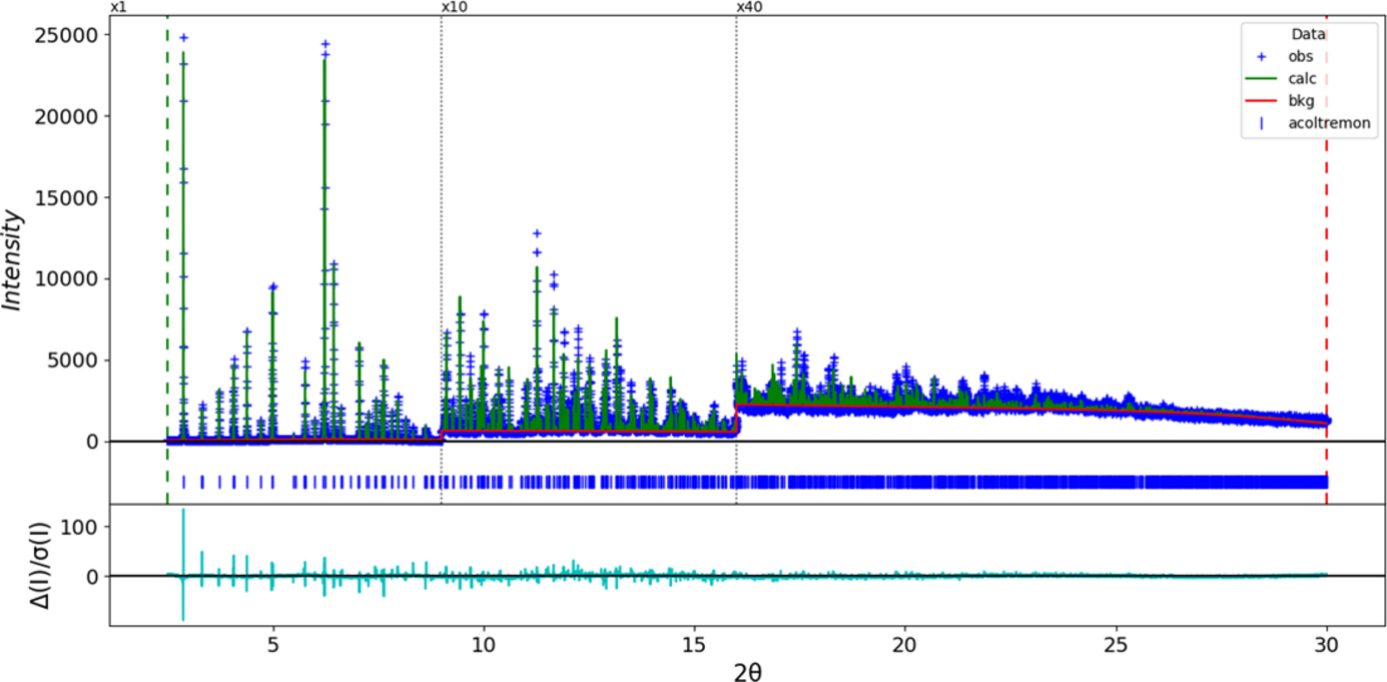
The Rietveld plot for acoltremon. The blue crosses represent the observed data points, and the green line is the calculated pattern. The cyan curve is the normalized error plot, and the red line is the background curve. The blue tick marks indicate the peak positions. The vertical scale has been multiplied by a factor of 10× for 2θ > 9.0° and by a factor of 40× for 2θ > 16.0°.

The volume enclosed by the Hirshfeld surface of acoltremon (Fig. 9 ▸; Spackman *et al.*, 2021 ▸) is 424.36 Å^3^, 98.31% of 1/4 of the unit-cell volume. The packing density is thus typical. The only significant close contacts (red in Fig. 8 ▸) involve the hydrogen bonds. The volume/non-hydrogen atom is larger than normal, at 20.5 Å^3^.

The Bravais–Friedel–Donnay–Harker (Bravais, 1866 ▸; Friedel, 1907 ▸; Donnay & Harker, 1937 ▸) algorithm suggests that we might expect isotropic morphology for acoltremon. A second-order spherical harmonic model for preferred orientation was included. The texture index was 1.034, indicating that the preferred orientation was slight in this rotated capillary specimen.

## 4. Database survey

A reduced cell search in the Cambridge Structural Database (CSD 2026.1.0; Groom *et al.*, 2016 ▸), combined with the chemistry C, H, N, and O only, yielded 22 hits, but no structures for acoltremon or its derivatives.

## 5. Synthesis and crystallization

Acoltremon is a commercial reagent and was purchased from TargetMol (Batch #141432) and used as-received.

## 6. Refinement

Crystal data, data collection and structure refinement details are summarized in Table 3 ▸. The white powder was packed into a 1.5 mm diameter Kapton capillary, and rotated during the measurement at ∼50 Hz. The powder pattern was measured at 295 K at beam line 11-BM (Lee *et al.*, 2008 ▸; Wang *et al.*, 2008 ▸; Antao *et al.*, 2008 ▸) of the Advanced Photon Source at Argonne National Laboratory using a wavelength of 0.4687342 Å from 0.5–50° 2θ with a step size of 0.001° and a counting time of 0.1 sec step^−1^. The high-resolution powder diffraction data were collected using twelve silicon crystal analyzers that allow for high angular resolution, high precision, and accurate peak positions. A mixture of silicon (NIST SRM 640c) and alumina (NIST SRM 676a) standards (ratio Al_2_O_3_:Si = 2:1 by weight) was used to calibrate the instrument and refine the monochromatic wavelength used in the experiment.

The pattern was indexed on a primitive ortho­rhom­bic unit cell with *a* = 9.32059, *b* = 11.39187, *c* = 16.25977 Å, *V* = 1727.5 Å^3^, and *Z* = 4 using *N-TREOR* as incorporated into *EXPO2014* (Altomare *et al.*, 2013 ▸). The suggested space group was *P*2_1_2_1_2_1_, which was confirmed by successful solution and refinement of the structure.

The *a*, *b*, and *c* lattice parameters at 298 K were 2.0% larger, 9.7% larger, and 7.0% smaller than those reported at 100 K. Refinement was started using the fractional coordinates of Rodriguez-Arévalo *et al.* (2021 ▸), before we realized that they were for a different diastereomer. The refinement changed the chiralities to result in the enanti­omer of the correct diastereomer.

To make a cleaner narrative, the mol­ecular structure of (1*R*,2*S*,5*R*)-acoltremon was downloaded from PubChem (Kim *et al.*, 2023 ▸) as Conformer3D_COMPOUND_CID_11266244.sdf. It was converted to a *.mol2 file using *Mercury* (Macrae *et al.*, 2020 ▸). The structure was solved using Monte Carlo simulated annealing techniques as implemented in *EXPO2014* (Altomare *et al.*, 2013 ▸).

Rietveld refinement was carried out using *GSAS-II* (Toby & Von Dreele, 2013 ▸). Only the 2.5–30.0° portion of the pattern was included in the refinements (*d_min_* = 0.905 Å). The μ*R* value was fixed at 0.00, calculated using the 11-BM website (https://11bm.xray.aps.anl.gov/absorb/). All non-H bond distances and angles were subjected to restraints, based on a *Mercury* Mogul geometry check (Sykes *et al.*, 2011 ▸; Bruno *et al.*, 2004 ▸). The Mogul average and standard deviation for each qu­antity were used as the restraint parameters. The aromatic ring was restrained to be planar. The restraints contributed 4.0% to the overall *χ^2^*. The hydrogen atoms were included in calculated positions, which were recalculated during the refinement using *Materials Studio* (Dassault Systèmes, 2024 ▸). The *U*_iso_(H) values were grouped by chemical similarity. The peak profiles were described using an isotropic microstrain model, with the strain fixed at 10 ppm. The background was modeled using a six-term shifted Chebyshev polynomial, with a peak at 6.17° to model the scattering from the Kapton capillary and any amorphous component of the sample.

The final refinement of 83 variables using 27,501 observations and 53 restraints yielded the residuals *R*_wp_ = 0.1352 and GOF = 3.59. The largest peak (0.13 Å from C15) and hole (1.19 Å from C10) in the difference Fourier map are 1.07 (16) and −0.67 (16) e Å^−3^, respectively. The final Rietveld plot is shown in Fig. 9 ▸. The largest features in the normalized error plot are in the positions and shapes of some of the strong low-angle peaks, and may indicate a change in the specimen during the measurement.

The crystal structures of both diastereomers were optimized (fixed experimental unit cells) with density functional theory techniques using *VASP* (Kresse & Furthmüller, 1996 ▸) through the *MedeA* graphical inter­face (Materials Design, 2024 ▸). The calculations were carried out on 32 cores of a 144-core (768 Gb memory) HPE Superdome Flex 280 Linux server at North Central College. The calculation used the GGA-PBE functional, a plane wave cutoff energy of 400.0 eV, and a *k*-point spacing of 0.5 Å^−1^ leading to a 2 × 2 × 1 mesh. To permit comparison of the energies and lattice parameters of the two diastereomers, dispersion-corrected DFT calculations were also carried out using *VASP*, incorporating the DFT-D3 approach of Grimme and allowing the lattice parameters to optimize. Single-point density functional theory calculations (fixed experimental cell) and population analysis were carried out using *CRYSTAL23* (Erba *et al.*, 2023 ▸). (fixed experimental cell) and population analysis were carried out using *CRYSTAL17* (Dovesi *et al.*, 2018 ▸). The basis sets for the H, C, N and O atoms in the calculation were those of Gatti *et al.* (1994 ▸). The calculations were run on a 3.5 GHz PC using 8 *k*-points and the B3LYP functional.

## Supplementary Material

Crystal structure: contains datablock(s) acoltremon_2, Molecules_100K_VASP, acoltremon_2_VASP. DOI: 10.1107/S2056989026006572/hb8218sup1.cif

Supporting information file. DOI: 10.1107/S2056989026006572/hb8218acoltremon_2sup2.cml

Table 5, with experimental and calculated lattice parameters for the two diastereomers. DOI: 10.1107/S2056989026006572/hb8218sup3.docx

CCDC references: 2564140, 2564139, 2564138

Additional supporting information:  crystallographic information; 3D view; checkCIF report

## Figures and Tables

**Table 1:** Hydrogen-bond geometry (Å, °) for *VASP*-optimized acoltremon

*D*—H⋯*A*	*D*—H	H⋯*A*	*D*⋯*A*	*D*—H⋯*A*	Mulliken overlap	H-bond energy
N3—H41⋯O1^i^	1.04	1.80	2.836	175	0.064	5.8
C5—H23⋯O1^i^	1.10	2.47	3.428	145	0.014	–
C13—H35⋯O2	1.10	2.61	3.594	148	0.010	–
C10—H31⋯C11^ii^	1.11	2.50	2.991	106	0.012	–

Symmetry codes: (i) −*x* − 1, *y* + 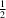, −*z* + 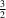; (ii) *x* + 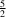, −*y* − 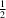, −*z* + 1.

**Table 2:** Hydrogen-bond geometry (Å, °) for *VASP*-optimized (1*S*,2*S*,5*R*) diastereomer

*D*—H⋯*A*	*D*—H	H⋯*A*	*D*⋯*A*	*D*—H⋯*A*	Mulliken overlap	H-bond energy
N1—H1*N*⋯O1^i^	1.03	1.89	2.922	176	0.048	5.1
C18—H18*B*⋯O1^ii^	1.10	2.30	3.383	169	0.022	
C10—H10⋯O1^iii^	1.10	2.48	3.466	149	0.016	
C9–H9*B*⋯O2	1.10	2.61	3.526	140	0.011	
C3–H00*F*⋯C11	1.11	2.55	2.963	101	0.011	

Symmetry codes: (i) −*x* + 1, *y* + 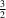, −*z* + 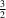; (ii) *x* + 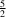, −*y* − 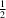, −*z* + 1; (iii) −*x* − 1, *y* + 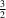, −*z* + 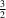.

**Table 3:** Experimental details

	acoltremon_2
Crystal data	
Chemical formula	C_18_H_27_NO_2_
*M*_r_	289.42
Crystal system, space group	Orthorhombic, *P*2_1_2_1_2_1_
Temperature (K)	295
*a*, *b*, *c* (Å)	9.320220 (15), 11.39111 (3), 16.26284 (4)
*V* (Å^3^)	1726.59 (1)
*Z*	4
Radiation type	Synchrotron, λ = 0.46873 Å
μ (mm^−1^)	0.005
Specimen shape, size (mm)	Cylinder, 2.0 × 1.5

Data collection	
Diffractometer	11-BM, APS
Specimen mounting	Kapton capillary
Data collection mode	Transmission
Scan method	Step
2θ values (°)	2θ_min_ = 0.510, 2θ_max_ = 49.995, 2θ_step_ = 0.001

Refinement	
*R* factors and goodness of fit	*R*_p_ = 0.113, *R*_wp_ = 0.133, *R*_exp_ = 0.038, *R*(*F*^2^) = 0.10052, χ^2^ = 12.888
No. of parameters	83
No. of restraints	53
(Δ/σ)_max_	1.712

Computer programs: *GSAS-II* (Toby & Von Dreele, 2013 ▸) and *DIAMOND* (Brandenburg & Putz, 2025 ▸).

## supplementary crystallographic information

### (1R,2S,5R)-2-Isopropyl-N-(4-methoxyphenyl)-5-methylcyclohexane-1-carboxamide (acoltremon_2) Crystal data

**Table:**

C_18_H_27_NO_2_	*V* = 1726.59 (1) Å^3^
*M**_r_* = 289.42	*Z* = 4
Orthorhombic, *P*2_1_2_1_2_1_	*D*_x_ = 1.113 Mg m^−^^3^
*a* = 9.320220 (15) Å	Synchrotron radiation, λ = 0.46873 Å
*b* = 11.39111 (3) Å	*T* = 295 K
*c* = 16.26284 (4) Å	cylinder, 2.0 × 1.5 mm

### (1R,2S,5R)-2-Isopropyl-N-(4-methoxyphenyl)-5-methylcyclohexane-1-carboxamide (acoltremon_2) Data collection

**Table:**

11-BM, APS diffractometer	Scan method: step
Specimen mounting: Kapton capillary	2θ_min_ = 0.510°, 2θ_max_ = 49.995°, 2θ_step_ = 0.001°
Data collection mode: transmission	

### (1R,2S,5R)-2-Isopropyl-N-(4-methoxyphenyl)-5-methylcyclohexane-1-carboxamide (acoltremon_2) Refinement

**Table:**

Least-squares matrix: full	83 parameters
*R*_p_ = 0.113	53 restraints
*R*_wp_ = 0.133	16 constraints
*R*_exp_ = 0.038	Weighting scheme based on measured s.u.'s
*R*(*F*^2^) = 0.10052	(Δ/σ)_max_ = 1.712
49486 data points	Background function: Background function: "chebyschev-1" function with 6 terms: 47.29(16), -6.65(24), -12.59(16), 2.79(16), -3.99(17), -0.00(16), Background peak parameters: pos, int, sig, gam: 6.167(16), 1.122(32)e4, 1.24(5)e4, 0.100,
Profile function: Finger-Cox-Jephcoat function parameters U, V, W, X, Y, SH/L: peak variance(Gauss) = Utan(Th)^2^+Vtan(Th)+W: peak HW(Lorentz) = X/cos(Th)+Ytan(Th); SH/L = S/L+H/L U, V, W in (centideg)^2^, X & Y in centideg 2.543, -0.174, 0.052, 0.000, 0.000, 0.002,	Preferred orientation correction: Simple spherical harmonic correction Order = 2 Coefficients: 0:0:C(2,0) = 0.290(5); 0:0:C(2,2) = 0.295(3)

### (1R,2S,5R)-2-Isopropyl-N-(4-methoxyphenyl)-5-methylcyclohexane-1-carboxamide (acoltremon_2) Fractional atomic coordinates and isotropic or equivalent isotropic displacement parameters (Å^2^)

**Table:**

	*x*	*y*	*z*	*U*_iso_*/*U*_eq_	
O1	1.0060 (5)	0.3097 (4)	0.5123 (3)	0.0774 (13)*	
O2	0.9044 (5)	0.1119 (5)	0.8745 (3)	0.122 (2)*	
N3	0.7965 (5)	0.2314 (5)	0.5481 (3)	0.0774 (13)*	
C4	0.8275 (6)	0.4484 (4)	0.4087 (3)	0.0785 (11)*	
C5	0.8154 (5)	0.3208 (4)	0.4181 (3)	0.0785 (11)*	
C6	0.7586 (6)	0.4837 (5)	0.3279 (4)	0.0785 (11)*	
C7	0.8365 (6)	0.2953 (5)	0.2633 (3)	0.0785 (11)*	
C8	0.8992 (6)	0.2713 (5)	0.3472 (4)	0.0785 (11)*	
C9	0.8316 (7)	0.4299 (6)	0.2550 (3)	0.0785 (11)*	
C10	0.7665 (7)	0.5165 (6)	0.4855 (4)	0.124 (2)*	
C11	0.8833 (5)	0.2836 (5)	0.4956 (3)	0.0774 (13)*	
C12	0.9335 (7)	0.2360 (6)	0.1913 (3)	0.0785 (11)*	
C13	0.7878 (7)	0.6442 (7)	0.4737 (4)	0.124 (2)*	
C14	0.5992 (7)	0.5117 (7)	0.4841 (5)	0.124 (2)*	
C15	0.8281 (7)	0.2023 (6)	0.6291 (3)	0.0637 (11)*	
C16	0.7831 (7)	0.0934 (5)	0.6598 (3)	0.0637 (11)*	
C17	0.8947 (7)	0.2790 (4)	0.6808 (4)	0.0637 (11)*	
C18	0.8106 (7)	0.0608 (5)	0.7419 (4)	0.0637 (11)*	
C19	0.9405 (6)	0.2401 (5)	0.7600 (4)	0.0637 (11)*	
C20	0.8859 (7)	0.1368 (6)	0.7939 (3)	0.0637 (11)*	
C21	0.8311 (7)	0.0201 (7)	0.9119 (4)	0.122 (2)*	
H22	0.94662	0.47012	0.40403	0.0942*	
H23	0.69849	0.29161	0.41551	0.0942*	
H24	0.76261	0.58335	0.32187	0.0942*	
H25	0.64173	0.45417	0.32732	0.0942*	
H26	0.72280	0.25865	0.26023	0.0942*	
H27	1.01186	0.31027	0.34713	0.0942*	
H28	0.90827	0.17223	0.35567	0.0942*	
H29	0.77023	0.45365	0.19669	0.0942*	
H30	0.94572	0.46530	0.25046	0.0942*	
H31	0.81334	0.48356	0.54600	0.1482*	
H32	1.05097	0.26012	0.20108	0.0942*	
H33	0.92100	0.13649	0.19352	0.0942*	
H34	0.89651	0.26980	0.12882	0.0942*	
H35	0.68069	0.69145	0.48081	0.1482*	
H36	0.83250	0.66107	0.40953	0.1482*	
H37	0.86659	0.67839	0.52166	0.1482*	
H38	0.56192	0.41944	0.50089	0.1482*	
H39	0.55441	0.57720	0.53046	0.1482*	
H40	0.55903	0.53493	0.41992	0.1482*	
H41	0.69380	0.20820	0.53000	0.0928*	
H42	0.72320	0.02953	0.61815	0.0764*	
H43	0.91356	0.37362	0.66070	0.0764*	
H44	0.77148	−0.02757	0.76598	0.0764*	
H45	1.02243	0.29354	0.79616	0.0764*	
H46	0.91141	−0.04652	0.93674	0.1460*	
H47	0.75825	−0.02427	0.86494	0.1460*	
H48	0.76348	0.05576	0.96470	0.1460*	

### (1R,2S,5R)-2-Isopropyl-N-(4-methoxyphenyl)-5-methylcyclohexane-1-carboxamide (acoltremon_2) Geometric parameters (Å, º)

**Table:**

O1—C11	1.213 (4)	C15—N3	1.391 (4)
O2—C20	1.352 (5)	C15—C16	1.402 (5)
O2—C21	1.389 (7)	C15—C17	1.362 (4)
N3—C11	1.317 (5)	C16—C15	1.402 (5)
N3—C15	1.391 (4)	C16—C18	1.410 (4)
N3—H41	1.035 (4)	C16—H42	1.140 (4)
C4—C5	1.465 (5)	C17—C15	1.362 (4)
C4—C6	1.517 (5)	C17—C19	1.428 (5)
C4—C10	1.575 (5)	C17—H43	1.139 (4)
C4—H22	1.140 (5)	C18—C16	1.410 (4)
C5—C4	1.465 (5)	C18—C20	1.399 (5)
C5—C8	1.502 (4)	C18—H44	1.140 (4)
C5—C11	1.472 (4)	C19—C17	1.428 (5)
C5—H23	1.140 (5)	C19—C20	1.395 (5)
C6—C4	1.517 (5)	C19—H45	1.140 (4)
C6—C9	1.498 (5)	C20—O2	1.352 (5)
C6—H24	1.141 (6)	C20—C18	1.399 (5)
C6—H25	1.140 (6)	C20—C19	1.395 (5)
C7—C8	1.509 (6)	C21—O2	1.389 (7)
C7—C9	1.540 (6)	C21—H46	1.139 (8)
C7—C12	1.626 (6)	C21—H47	1.140 (7)
C7—H26	1.140 (6)	C21—H48	1.140 (7)
C8—C5	1.502 (4)	H22—C4	1.140 (5)
C8—C7	1.509 (6)	H23—C5	1.140 (5)
C8—H27	1.140 (6)	H24—C6	1.141 (6)
C8—H28	1.140 (6)	H25—C6	1.140 (6)
C9—C6	1.498 (5)	H26—C7	1.140 (6)
C9—C7	1.540 (6)	H27—C8	1.140 (6)
C9—H29	1.140 (5)	H28—C8	1.140 (6)
C9—H30	1.140 (6)	H29—C9	1.140 (5)
C10—C4	1.575 (5)	H30—C9	1.140 (6)
C10—C13	1.481 (8)	H31—C10	1.140 (6)
C10—C14	1.561 (6)	H32—C12	1.140 (7)
C10—H31	1.140 (6)	H33—C12	1.140 (7)
C11—O1	1.213 (4)	H34—C12	1.140 (6)
C11—N3	1.317 (5)	H35—C13	1.140 (7)
C11—C5	1.472 (4)	H36—C13	1.140 (7)
C12—C7	1.626 (6)	H37—C13	1.140 (7)
C12—H32	1.140 (7)	H38—C14	1.140 (7)
C12—H33	1.140 (7)	H39—C14	1.140 (7)
C12—H34	1.140 (6)	H40—C14	1.140 (7)
C13—C10	1.481 (8)	H41—N3	1.035 (4)
C13—H35	1.140 (7)	H42—C16	1.140 (4)
C13—H36	1.140 (7)	H43—C17	1.139 (4)
C13—H37	1.140 (7)	H44—C18	1.140 (4)
C14—C10	1.561 (6)	H45—C19	1.140 (4)
C14—H38	1.140 (7)	H46—C21	1.139 (8)
C14—H39	1.140 (7)	H47—C21	1.140 (7)
C14—H40	1.140 (7)	H48—C21	1.140 (7)
			
C20—O2—C21	121.3 (5)	H32—C12—H33	109.5 (5)
C11—N3—C15	126.2 (4)	H32—C12—H34	109.5 (5)
C11—N3—H41	119.9 (4)	H33—C12—H34	109.4 (5)
C15—N3—H41	113.8 (5)	C10—C13—H35	109.4 (6)
C5—C4—C6	108.7 (4)	C10—C13—H36	109.4 (6)
C5—C4—H22	107.3 (4)	H35—C13—H36	109.5 (6)
C6—C4—H22	107.3 (5)	C10—C13—H37	109.5 (7)
C4—C5—C8	104.6 (4)	H35—C13—H37	109.5 (6)
C4—C5—C11	110.0 (3)	H36—C13—H37	109.5 (5)
C8—C5—C11	109.0 (4)	C10—C14—H38	109.4 (6)
C4—C5—H23	111.1 (4)	C10—C14—H39	109.5 (6)
C8—C5—H23	111.0 (4)	H38—C14—H39	109.5 (6)
C11—C5—H23	111.0 (4)	C10—C14—H40	109.4 (5)
C4—C6—C9	112.7 (4)	H38—C14—H40	109.5 (6)
C4—C6—H24	108.9 (5)	H39—C14—H40	109.5 (6)
C9—C6—H24	108.9 (5)	N3—C15—C16	119.0 (5)
C4—C6—H25	109.5 (5)	N3—C15—C17	121.9 (5)
C9—C6—H25	107.9 (5)	C16—C15—C17	119.0 (3)
H24—C6—H25	108.9 (4)	C15—C16—C18	121.0 (3)
C8—C7—C9	105.7 (4)	C15—C16—H42	120.0 (5)
C8—C7—H26	109.5 (5)	C18—C16—H42	119.0 (5)
C9—C7—H26	109.4 (5)	C15—C17—C19	119.6 (3)
C5—C8—C7	115.1 (4)	C15—C17—H43	120.0 (5)
C5—C8—H27	109.5 (5)	C19—C17—H43	120.4 (5)
C7—C8—H27	106.5 (5)	C16—C18—C20	120.1 (3)
C5—C8—H28	108.5 (5)	C16—C18—H44	119.9 (5)
C7—C8—H28	108.4 (5)	C20—C18—H44	120.0 (5)
H27—C8—H28	108.5 (4)	C17—C19—C20	120.6 (3)
C6—C9—C7	110.5 (4)	C17—C19—H45	120.0 (6)
C6—C9—H29	109.5 (5)	C20—C19—H45	119.4 (6)
C7—C9—H29	108.9 (5)	O2—C20—C18	121.3 (5)
C6—C9—H30	109.3 (6)	O2—C20—C19	120.9 (5)
C7—C9—H30	109.3 (5)	C18—C20—C19	117.8 (3)
H29—C9—H30	109.3 (4)	O2—C21—H46	109.5 (6)
C13—C10—C14	99.6 (5)	O2—C21—H47	109.4 (5)
C13—C10—H31	112.5 (6)	H46—C21—H47	109.5 (7)
C14—C10—H31	112.5 (6)	O2—C21—H48	109.4 (7)
O1—C11—N3	123.0 (4)	H46—C21—H48	109.5 (5)
O1—C11—C5	121.7 (3)	H47—C21—H48	109.5 (6)
N3—C11—C5	114.9 (3)		

### (acoltremon_2_VASP) Crystal data

**Table:**

C_18_H_27_NO_2_	*b* = 11.39111 Å
*M**_r_* = 289.42	*c* = 16.26284 Å
Orthorhombic, *P*2_1_2_1_2_1_	*V* = 1726.59 Å^3^
*a* = 9.32022 Å	*Z* = 4

### (acoltremon_2_VASP) Data collection

**Table:**

*h* = →	*l* = →
*k* = →	

### (acoltremon_2_VASP) Fractional atomic coordinates and isotropic or equivalent isotropic displacement parameters (Å^2^)

**Table:**

	*x*	*y*	*z*	*B*_iso_*/*B*_eq_	
O1	1.01413	0.30651	0.51100		
O2	0.90354	0.11941	0.87778		
N3	0.79574	0.23233	0.54912		
C4	0.82622	0.46054	0.40507		
C5	0.81815	0.32519	0.41433		
C6	0.76351	0.49545	0.32138		
C7	0.84080	0.30145	0.25810		
C8	0.89693	0.26450	0.34276		
C9	0.84152	0.43556	0.25010		
C10	0.76145	0.52847	0.47885		
C11	0.88472	0.28716	0.49524		
C12	0.92763	0.24298	0.18986		
C13	0.80650	0.65778	0.47776		
C14	0.59804	0.51763	0.48653		
C15	0.83074	0.19945	0.63072		
C16	0.77289	0.09627	0.66346		
C17	0.91421	0.27182	0.68170		
C18	0.79465	0.06595	0.74578		
C19	0.93765	0.24165	0.76324		
C20	0.87726	0.13914	0.79624		
C21	0.81861	0.03231	0.91816		
H22	0.94183	0.48270	0.40418		
H23	0.70449	0.29852	0.41349		
H24	0.76879	0.59126	0.31368		
H25	0.64871	0.47176	0.31927		
H26	0.72797	0.27240	0.25317		
H27	1.01212	0.28588	0.34672		
H28	0.88899	0.16829	0.34954		
H29	0.79251	0.46137	0.19103		
H30	0.95403	0.46632	0.24810		
H31	0.80890	0.48978	0.53495		
H32	1.04086	0.27042	0.19257		
H33	0.92576	0.14661	0.19508		
H34	0.88640	0.26639	0.12866		
H35	0.75606	0.70558	0.42664		
H36	0.92354	0.66743	0.47264		
H37	0.77364	0.70176	0.53494		
H38	0.56228	0.42571	0.48786		
H39	0.56132	0.55978	0.54368		
H40	0.54279	0.56176	0.43530		
H41	0.69185	0.21534	0.52992		
H42	0.70813	0.03924	0.62428		
H43	0.95775	0.35348	0.65753		
H44	0.74655	−0.01411	0.76994		
H45	0.99958	0.29888	0.80367		
H46	0.84877	0.03673	0.98317		
H47	0.84234	−0.05661	0.89556		
H48	0.70326	0.05132	0.91125		

### (acoltremon_2_VASP) Hydrogen-bond geometry (Å, º)

**Table:**

*D*—H···*A*	*D*—H	H···*A*	*D*···*A*	*D*—H···*A*
N3—H41···O1^i^	1.04	1.80	2.836	175
C5—H23···O1^i^	1.10	2.47	3.428	145
C13—H35···O2	1.10	2.61	3.594	148
C10—H31···C11^ii^	1.11	2.50	2.991	106

Symmetry codes: (i) −*x*−1, *y*+1/2, −*z*+3/2; (ii) *x*+5/2, −*y*−1/2, −*z*+1.

### (Molecules_100K_VASP) Crystal data

**Table:**

C_18_H_27_NO_2_	α = 90°
*M**_r_* = 289.42	β = 90°
*P*2_1_2_1_2_1_	γ = 90°
*a* = 9.13710 Å	*V* = 1659.07 Å^3^
*b* = 10.38210 Å	*Z* = 4
*c* = 17.48930 Å	

### (Molecules_100K_VASP) Data collection

**Table:**

*h* = →	*l* = →
*k* = →	

### (Molecules_100K_VASP) Fractional atomic coordinates and isotropic or equivalent isotropic displacement parameters (Å^2^)

**Table:**

	*x*	*y*	*z*	*U*_iso_*/*U*_eq_	
O1	0.8885	0.6624	0.5105	?	
O2	0.9034	0.0242	0.8026	?	
N1	0.6836	0.7769	0.5428	?	
C1	0.4621	0.4887	0.5510	?	
C2	0.6165	0.2918	0.5384	?	
C3	0.6132	0.4381	0.5276	?	
C4	0.6568	0.4789	0.4455	?	
C5	0.7971	0.4126	0.4174	?	
C6	0.8423	0.4550	0.3372	?	
C7	0.8627	0.6007	0.3317	?	
C8	0.9099	0.6428	0.2520	?	
C9	0.7217	0.6676	0.3564	?	
C10	0.6655	0.6279	0.4362	?	
C11	0.7566	0.6895	0.4990	?	
C12	0.7420	0.8372	0.6090	?	
C13	0.8065	0.7644	0.6670	?	
C14	0.8608	0.8232	0.7329	?	
C15	0.8508	0.9571	0.7411	?	
C16	0.7842	0.0306	0.6835	?	
C17	0.7303	0.9709	0.6182	?	
C18	0.9601	0.9513	0.8652	?	
H1N	0.5786	0.8009	0.5264	?	
H1A	0.4530	0.5939	0.5468	?	
H1B	0.4372	0.4617	0.6104	?	
H1C	0.3768	0.4467	0.5142	?	
H2A	0.5461	0.2438	0.4959	?	
H2B	0.5744	0.2658	0.5953	?	
H2C	0.7273	0.2517	0.5331	?	
H00F	0.6953	0.4796	0.5671	?	
H4	0.5669	0.4483	0.4070	?	
H5A	0.8866	0.4336	0.4578	?	
H5B	0.7817	0.3074	0.4178	?	
H6A	0.9438	0.4055	0.3201	?	
H6B	0.7577	0.4254	0.2955	?	
H7	0.9499	0.6281	0.3720	?	
H8A	0.8272	0.6157	0.2090	?	
H8B	0.0141	0.5972	0.2357	?	
H8C	0.9255	0.7478	0.2491	?	
H9A	0.6348	0.6435	0.3149	?	
H9B	0.7337	0.7731	0.3539	?	
H10	0.5539	0.6665	0.4413	?	
H13	0.8143	0.6603	0.6610	?	
H14	0.9108	0.7635	0.7769	?	
H16	0.7757	0.1346	0.6910	?	
H17	0.6780	0.0285	0.5738	?	
H18A	0.8752	0.8875	0.8892	?	
H18B	0.9948	0.0218	0.9082	?	
H18C	0.0551	0.8926	0.8477	?	

### (Molecules_100K_VASP) Hydrogen-bond geometry (Å, º)

**Table:**

*D*—H···*A*	*D*—H	H···*A*	*D*···*A*	*D*—H···*A*
N1—H1*N*···O1	1.03	1.89	2.922	176
C18—H18*B*···O1	1.10	2.30	3.383	169
C10—H10···O1	1.10	2.48	3.466	149
C9—H9*B*···O2	1.10	2.61	3.526	140
C3—H00*F*···C11	1.11	2.55	2.963	101
