# Supplementary material for: Crystal structure of the (1R,2S,5R) diastereomer of acoltremon, C18H27NO2, from synchrotron powder diffraction data and density functional theory calculations
Source: Acta Crystallogr E Crystallogr Commun. 2026 Jun 26;82(Pt 7):877–82. doi: 10.1107/S2056989026006572 (PMC13330845; doi:10.1107/S2056989026006572)
Supplement: Supplementary file 3 [file e-82-00877-sup3.docx]

Table 5. Lattice parameters of acoltremon diastereomers. Space group *P2_1_2_1_2_1_*.

| Diastereomer | (1*S*,2*S*,5*R*)  Rodriguez-Arévalo et al., 2021 | | (1*R*,2*S*,5*R*)  This Work | |
| --- | --- | --- | --- | --- |
| T, K | 100 | DFT-D | 298 | DFT-D |
| *a*, Å | 9.1371(2) | 8.814820 | 9.320220(15) | 8.986659 |
| *b*, Å | 10.3821(3) | 10.034797 | 11.391105(27) | 10.739902 |
| *c*, Å | 17.4893(4) | 17.043063 | 16.26284(4) | 15.817871 |
| *V*, Å^3^ | 1659.08(7) | 1507.543 | 1726.587(5) | 1526.674 |
| E, eV/cell |  | -1149.036715 |  | -1150.045457 |
